# Trends in Influenza Vaccination Rates in Participants With Airflow Limitation: The Korea National Health and Nutrition Examination Survey 2007–2018

**DOI:** 10.3389/fmed.2022.870617

**Published:** 2022-05-03

**Authors:** Hyun Lee, Hayoung Choi, Yong Suk Jo

**Affiliations:** ^1^Division of Pulmonary Medicine and Allergy, Department of Internal Medicine, Hanyang University College of Medicine, Seoul, South Korea; ^2^Division of Pulmonary, Allergy, and Critical Care Medicine, Department of Internal Medicine, Hallym University Kangnam Sacred Heart Hospital, Hallym University College of Medicine, Seoul, South Korea; ^3^Division of Pulmonary and Critical Care Medicine, Department of Internal Medicine, Seoul St. Mary's Hospital, College of Medicine, The Catholic University of Korea, Seoul, South Korea

**Keywords:** influenza, vaccination, airflow limitation, risk factor, National Health and Nutrition Examination Survey (NHANES)

## Abstract

**Background:**

Influenza vaccination is strongly recommended for people with chronic lung diseases, including chronic obstructive pulmonary disease, to reduce risk of exacerbation. We assess the influenza vaccination rate and its related factors in participants with airflow limitation (AFL) using nationally representative data in Korea.

**Methods:**

We conducted a cross-sectional study from the Korea National Health and Nutrition Examination Survey from 2007 to 2018. Individuals ≥ 40 years who underwent spirometry and had identifiable information on influenza vaccination status were included.

**Results:**

Overall influenza vaccination coverage was 61.2% in participants with AFL and 41.8% in participants without AFL. Age had a significant impact on the yearly vaccination rate in participants with AFL. Over the 10 years of study period, while the yearly vaccination rate steadily increased from 58.3 to 61.9% in elderly participants (≥65 years) with AFL (*p* for trend = 0.117), the yearly vaccination rate decreased from 41.5% to 30.8% in younger participants (<65 years) (*p* for trend = 0.038). In multivariable analyses, younger age [adjusted odds ratio (OR) for unvaccinated = 0.88, 95% confidence interval (CI) = 0.87–0.90], male (adjusted OR = 1.64; 95% CI = 1.23–2.19), and current smokers (adjusted OR = 1.42, 95% CI = 1.01–2.00) were associated with increased odds of being unvaccinated.

**Conclusions:**

The vaccination rate in participants with AFL affected by age. Younger age, male sex, and current smoking were associated with unvaccinated status. More attention and targeted interventions are required to improve the influenza vaccination rate in those with AFL.

## Introduction

Chronic obstructive pulmonary disease (COPD), which is a representative disease with airflow limitation (AFL), is the leading causes of death worldwide, with a prevalence of 5.6% in 2015, which is expected to increase to 7.8% by 2030 (1, 2). COPD is a chronic inflammatory airway disease characterized by fixed airflow limitation and chronic respiratory symptoms, such as cough, sputum, and progressive dyspnea. Acute exacerbation of COPD (AECOPD) can occur during the natural course of disease (3). AECOPD not only affects an individual's physical health status but also decreases lung function and increases future risk of exacerbation and even mortality (4–6). AECOPD also increases medical expense and resource use, causing increased socioeconomic burden (7).

AECOPD is a heterogeneous event thought to be caused by complex interactions between the host, respiratory viruses or bacteria, and environmental pollution (8, 9). The most common causes of AECOPD are respiratory infections, most of which are viral. Although the most frequent viruses associated with exacerbation are human rhinoviruses (8), influenza is also important, accounting for up to 28% of COPD exacerbations (10). Influenza can lead to hospitalization, frequent exacerbation, and even death in patients with COPD (11, 12). The disease course of influenza is worse in patients with COPD compared with those without COPD.

Influenza vaccination is the main strategy for prevention and control of seasonal influenza (13). The influenza vaccination has been shown to reduce AECOPD, influenza-related hospitalization, and mortality (14, 15). Thus, seasonal influenza vaccination is recommended to stable COPD patients from groups A to D classified by combining exacerbation history and severity of dyspnea suggested by Global Initiative for Chronic Obstructive Lung Disease (GOLD) (16). Despite this recommendation, it is not known whether influenza vaccination coverage has increased among COPD patients because most studies evaluated vaccination rates for a certain year (17–20). Such information is even scarcer within the past 10 years (21).

In addition to COPD, influenza vaccination should be considered in relation to other respiratory diseases including asthma and bronchiectasis. A previous meta-analysis found that influenza vaccination reduced febrile illnesses and prevented asthma attacks requiring emergency visits and hospitalizations (22). Despite limited studies regarding the efficacy of influenza vaccination in bronchiectasis, international bronchiectasis guidelines strongly recommend influenza vaccination in patients with bronchiectasis (23, 24). Consequently, more studies are needed to evaluate the factors associated with influenza vaccination coverage among individuals with AFL to develop ways to encourage vaccination and improve their health outcomes.

In this study, we measure the influenza vaccination rate among participants with AFL in a nationally representative sample of Korea, and assess factors associated with influenza vaccination.

## Methods

### Study Population

For this study, cross-sectional data were used from the Korea National Health and Nutrition Examination Survey (KNHANES), which provides nationwide statistical data on the Korean population's health and diet from January 2007 to December 2018. The questionnaire on the influenza vaccination was investigated for only 3 months in 2013 and was not disclosed to the public; thus, data from 2013 were not included in this study. The KNHANES uses a complex, stratified, multistage probability cluster sampling design with sampling units of households based on geographic region, age, and gender. A health-related interview, nutrition survey, and physical examination were performed for each participant selected throughout Korea by trained interviewers. All participants agreed to participate in this study. Since publicly available data were used, ethical approval was waived. The KNHANES surveys were approved by the relevant institutional review boards, and informed consent was provided by all participants.

Because spirometry is only performed in individuals older than 40 years, we only included participants ≥40 years in the analyses. Participants who did not undergo spirometry and those who had missing data for influenza vaccination were excluded from the study.

**Figure 1:**
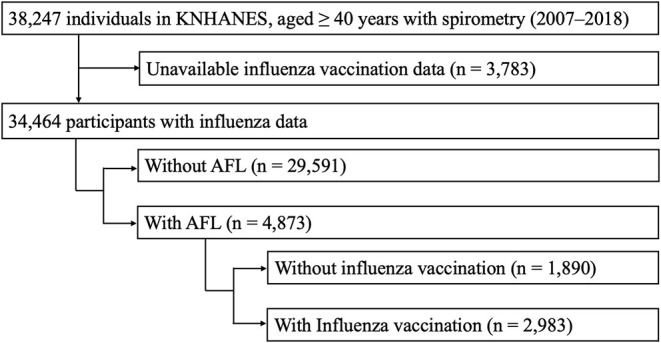
Selection of study population. AFL, airflow limitation; KNHANES, Korea National Health and Nutrition Examination Survey.

Of the total 38,247 participants, influenza vaccination data were available for 34,464, of whom 4,873 (14.1%) had AFL on spirometry and 29,591 (85.9%) did not. Finally, this study included 4,873 participants with AFL, comprising those without influenza vaccination (*n* = 1,890) and those with influenza vaccination (*n* = 2,983) (Figure 1).

### Exposure

The main exposure variable was influenza vaccination, which was determined by the response to a question about influenza vaccination status during the previous 1 year: “Have you been vaccinated for influenza during the previous 1 year?”

### Study Outcomes

The study outcomes were (1) influenza vaccination rate according to presence or absence of AFL during the study period; (2) influenza vaccination rate in participants with AFL stratified by age and AFL severity; and (3) factors associated with vaccination status in participants with AFL.

To reduce the disease burden of influenza, the Korean government instituted a national immunization program in 2005, which provides free influenza vaccination for people aged ≥ 65 years. Accordingly, we divided subjects into two age groups (65 years or older vs. under 65 years).

AFL was defined as spirometry revealing forced expiratory volume in 1 s (FEV_1_)/forced vital capacity (FVC) <0.7. Severity of AFL was classified according to the percentage of the predicted FEV_1_ (% pred): mild (FEV_1_ %pred of >70), moderate (FEV_1_ %pred of 60–69), moderately severe (FEV_1_ %pred of 50–59), severe (FEV_1_ %pred of 35–49), and very severe (FEV_1_ %pred of <35) (25).

### Covariates

The KNHANES provides various demographic data [age, sex, body mass index (BMI), education level, marital status, and self-perceived income status] and spirometry results. The Korean version of the EuroQol-5 dimensions questionnaire (EQ-5D), a simple health-related quality of life instrument consisting of 5 health dimensions (mobility, self-care, usual activities, pain/discomfort, and anxiety/depression), was used to measure QoL status (26, 27). Several comorbid conditions were also included in this study. The presence of hypertension was determined by high blood pressure (mean systolic blood pressure ≥140 mmHg or mean diastolic blood pressure ≥90 mmHg on examination or current intake of antihypertensive medications). Hypercholesterolemia was defined as total cholesterol >200 mg/dL or current intake of lipid-lowering medications (28). Diabetes was defined as fasting glucose >126 mg/dL or HbA1c >6.5% or current use of oral hypoglycemic agents or insulin for glycemic control (29). The presence of other comorbid conditions was determined by a positive response to the following two questions: “Have you been diagnosed with [disease] by a doctor?” or “Do you take medicine or treatment for [disease]?” (30).

### Statistical Analyses

Data are presented as medians and interquartile ranges for continuous variables and as frequencies (percentages) for categorical variables. Data were compared using the Mann–Whitney U test for continuous variables because of non-normality. Continuous variables were compared using Pearson's chi-squared test or Fisher's exact test, as appropriate. For the analyses of influenza vaccination rate by AFL severity and multivariable logistic regression to evaluate factors associated with not receiving influenza vaccination, we classified the severity of AFL into four groups [mild (FEV_1_ %pred of >70), moderate (FEV_1_ %pred of 60–69), moderately severe (FEV_1_ %pred of 50–59), and severe to very severe (FEV_1_ %pred of <50)] because there only 1.3% of participants (*n* = 64) had severe to very severe AFL (FEV_1_ %pred of <50) in the NHANES dataset. In the multivariable logistic regression model, we adjusted for age, sex, BMI, marital status, type of medical insurance, economic activity, education level, EQ-5D, chronic bronchitis, smoking history, the severity of AFL, comorbidities (hypertension, ischemic heart disease, diabetes mellitus, and asthma). All tests were two-sided, and a *p*-value ≤ 0.05 was considered statistically significant. Statistical analyses were performed using STATA software (ver. 16; StataCorp, College Station, TX, USA).

## Results

### Influenza Vaccination Rate According to Presence of Airflow Limitation

**Figure 2:**
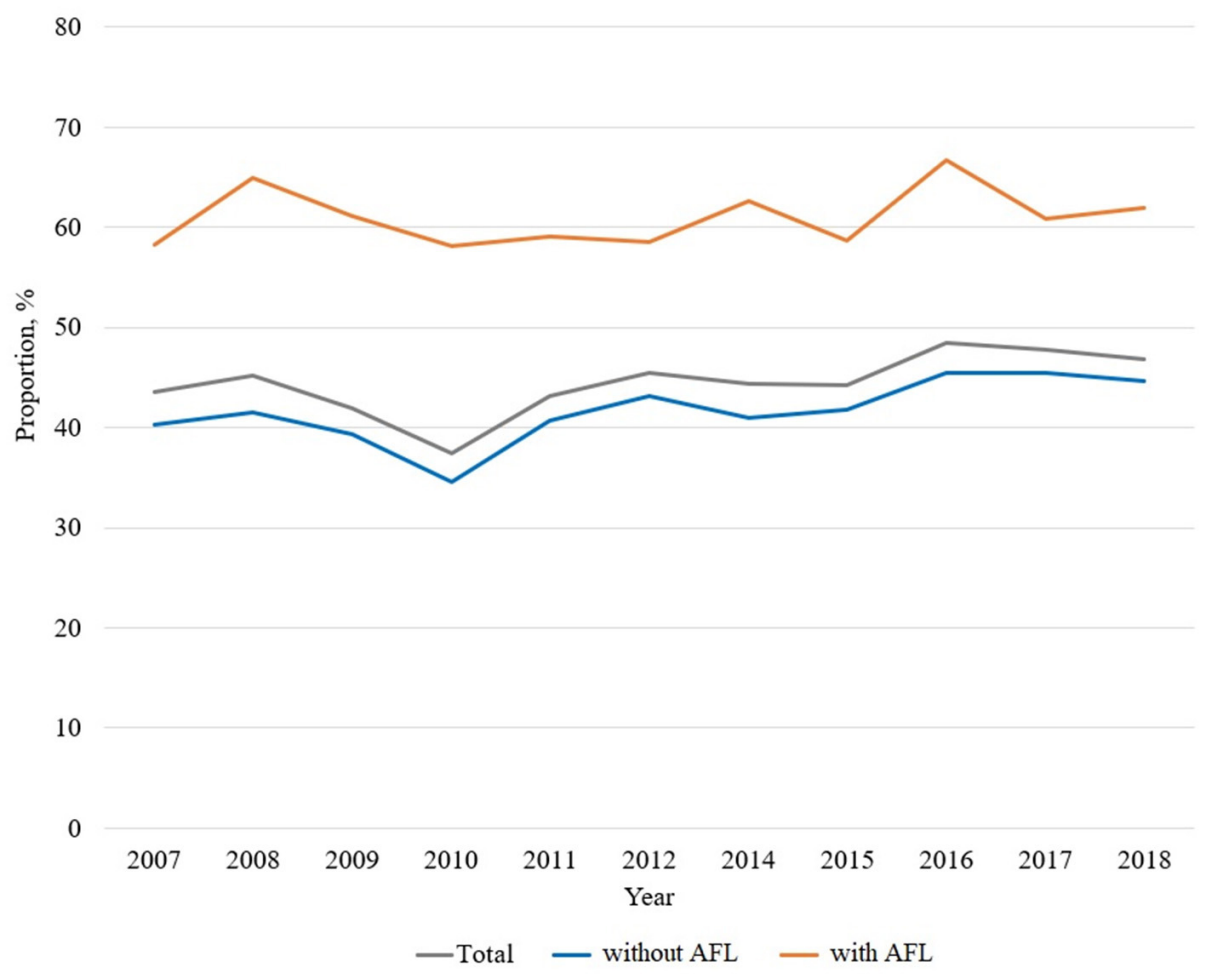
Influenza vaccination trends in participants with AFL. AFL, airflow limitation.

Influenza vaccination trends from 2007 to 2018 are shown in Figure 2. Compared to 61.2% of participants with AFL (2,983/4,873) being vaccinated, 41.8% participants without AFL (12,357/29,591) were vaccinated.

**Figure 3:**
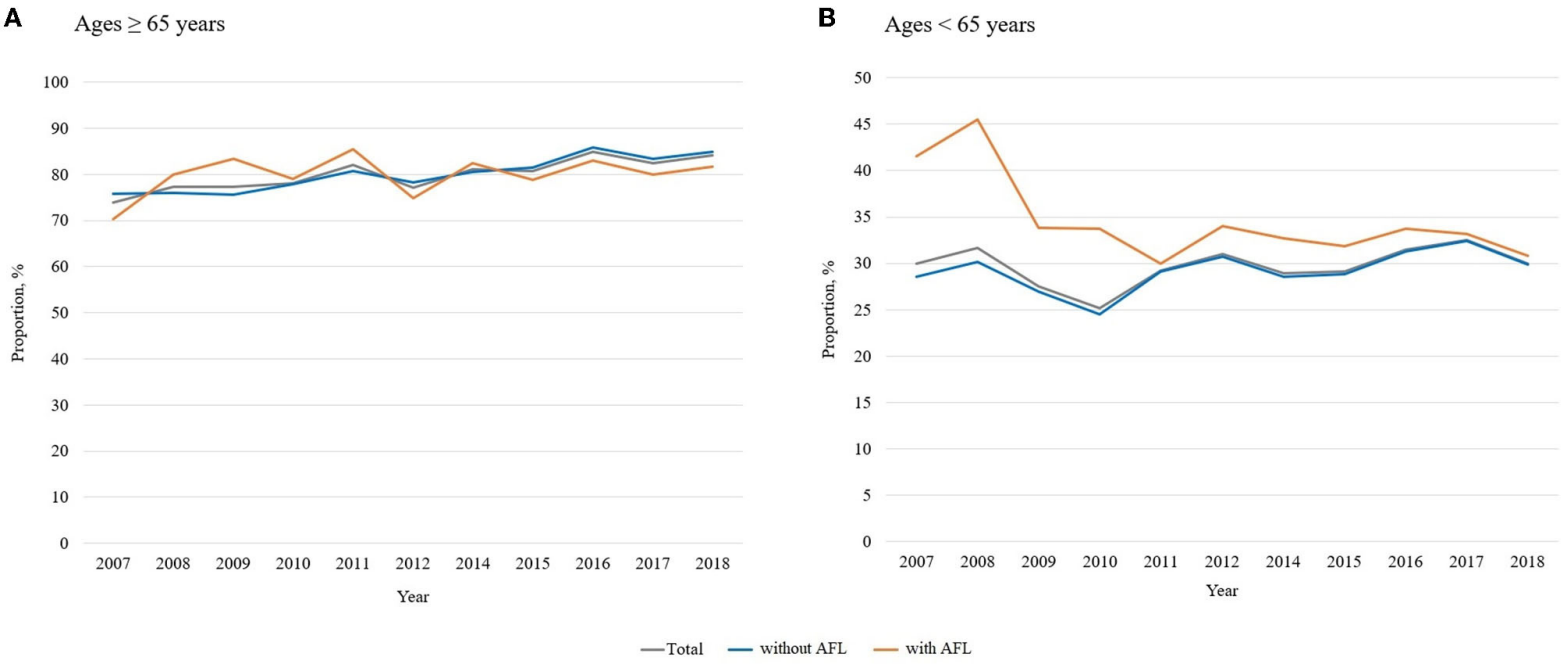
Influenza vaccination trends according to age (≥65 vs. <65 years). AFL, airflow limitation. **(A)** Age ≥ 65 years, **(B)** Age <65 years. AFL, airflow limitation.

Figure 3 shows the influenza vaccination trends according to AFL status based on age. While the overall vaccination rate in participants 65 years or older was ~80% (Figure 3A), the vaccination rate in participants younger than 65 years was <40% (Figure 3B). AFL had a significantly different impact on the age-stratified vaccination rate. In participants 65 years or older, the overall vaccination rate (80.2% with AFL vs. 80.3% without AFL; *p* = 0.894) was not different by AFL. Regardless of AFL, the yearly vaccination rate steadily increased during the study period [58.3% in 2007 to 61.9% in 2018 (*p* for trend = 0.117) in participants with AFL; 40.2% in 2007 to 44.7% in 2018 (*p* for trend < 0.001) in participants without AFL; Figure 3A].

However, among individuals 65 years or less, the vaccination rate was higher in participants with AFL than in those without AFL (34.7% with AFL vs. 29.2% without AFL; *p* < 0.001). Additionally, while there was a decreasing trend in the yearly vaccination rate among participants with AFL, the vaccination rate in subjects without AFL fluctuated [41.5% in 2007 to 30.8% in 2018 (*p* for trend = 0.038) for participants with AFL; 28.6% in 2007 to 29.9% in 2018 (*p* for trend < 0.001) in participants without AFL; Figure 3B].

### Comparison of Clinical Features in Participants With AFL According to Influenza Vaccination

Differences in clinical characteristics according to influenza vaccination status in participants with AFL are presented in Table 1. Vaccinated participants tended to be older and were less likely to be male and current smokers compared with unvaccinated participants (*p* < 0.001 for both). Regarding socioeconomics, vaccinated participants had lower economic activity and lower QoL compared with unvaccinated participants (*p* < 0.001 for both). Comorbid conditions including hypertension, ischemic heart disease, hypercholesterolemia, diabetes mellitus, cerebrovascular disease, asthma, and osteoporosis were more common among vaccinated participants than unvaccinated participants. Except for FEV_1_ % predicted, vaccinated participants had lower lung function parameters [FEV_1_ (L), FVC (L), FVC % pred, and FEV_1_/FVC] compared with unvaccinated participants (*p* < 0.001 for the latter four variables).

**Table 1:** Comparison of clinical features in participants with airflow limitation according to influenza vaccination.

	**Participants with airflow limitation (*****N*** **=** **4,873)**		
	**Without influenza vaccination** **(*n* = 1,890)**	**With influenza vaccination** **(*n* = 2,983)**	***P*-value**
Age (years)	60 (53–66)	70 (65–75)	<0.001
Male sex	1,449 (76.7)	2,061 (69.1)	<0.001
Current smoker	883 (47.1)	1,009 (34.1)	<0.001
Smoking amount (pack-years) (*n*=3,182)	5 (0–26)	0 (0–24.5)	0.004
Body mass index, kg/m^2^	23.5 (21.6–25.5)	23.5 (21.7–25.3)	0.782
Education beyond high school	1,107 (59.1)	1,149 (38.8)	<0.001
Marriage status (yes)	1,853 (98.2)	2,953 (99.2)	0.001
Economic activity status	1,257 (66.9)	1,352 (45.5)	<0.001
Health insurance	1,798 (95.8)	2,791 (94.4)	0.027
**Symptoms (*****n*** **=** **3,558)**			
Cough ≥3 months	45 (3.2)	88 (4.1)	0.207
Sputum ≥3 months	108 (7.8)	206 (9.5)	0.075
Chronic bronchitis	118 (8.5)	223 (10.3)	0.076
Quality of life, EQ-5D	1 (0.907–1)	1 (0.817–1)	<0.001
**Comorbidities**			
Hypertension (*n* = 3,576)	667 (51.5)	1,472 (64.5)	<0.001
Ischemic heart disease (*n* = 2,383)	53 (6.0)	152 (10.2)	<0.001
Hypercholesterolemia (*n* = 4,559)	298 (16.8)	571 (20.5)	0.002
Diabetes mellitus (*n* = 4,130)	853 (54.3)	1,512 (59.1)	0.003
Cerebrovascular disease (*n* = 2,354)	32 (3.6)	88 (6.0)	0.012
Asthma (*n* = 2,612)	63 (6.6)	200 (12.1)	<0.001
Osteoporosis (*n* = 1,958)	25 (3.5)	141 (11.4)	<0.001
Tuberculosis (*n* = 2,663)	49 (4.9)	87 (5.3)	0.633
Depression (*n* = 2,602)	26 (2.7)	61 (3.7)	0.151
Any cancer history^*^ (*n* = 1,361)	33 (3.8)	52 (3.5)	0.757
**Spirometry**			
FEV_1_, L	2.45 (1.96–2.90)	2.07 (1.60–2.49)	<0.001
FEV_1_, % pred	78.2 (68.6–86.6)	77.6 (66.5–87.6)	0.287
FVC, L	3.81 (3.12–4.44)	3.32 (2.66–3.90)	<0.001
FVC, % pred	91.9 (82.3–100.6)	87.2 (76.9–96.9)	<0.001
FEV_1_/FVC	66.0 (61.9–68.3)	65.0 (59.8–67.9)	<0.001

*Data are presented as median (interquartile range) or number (percentage). The numbers in brackets for each comorbidity represent the number of participants who responded to the questions regarding each comorbidity*.

* *Includes gastric, liver, colon, breast, cervical, lung, or thyroid cancer*.

*EQ-5D, EuroQol-5 dimensions questionnaire; FEV_1_, forced expiratory volume in 1s; % pred, percentage of the predicted value; FVC, forced vital capacity*.

### Influenza Vaccination Rate According to AFL Severity

**Figure 4:**
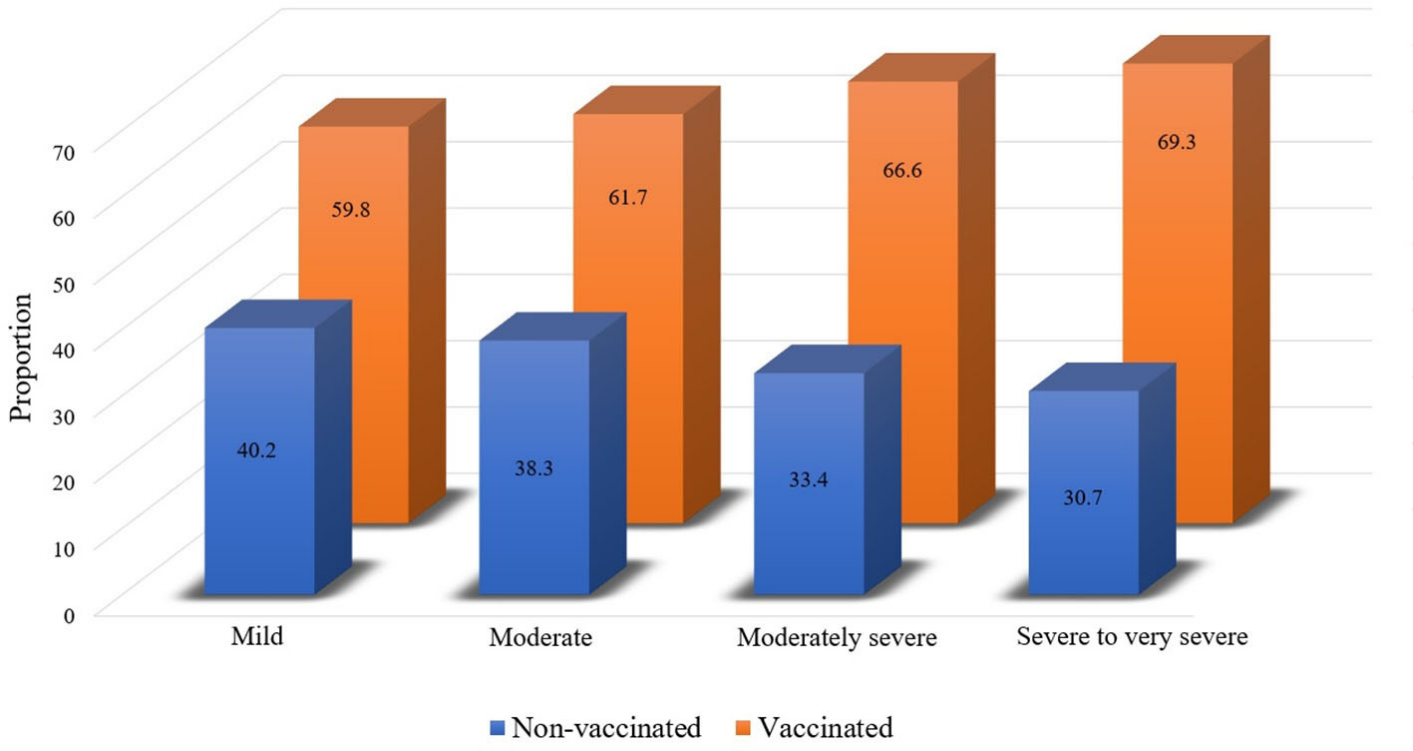
Influenza vaccination rate according to AFL severity. AFL, airflow limitation.

Influenza vaccination rate was further analyzed according to AFL severity. The overall vaccination rates were 59.8% (2,027/3,392), 61.7% (484/784), 66.6% (273/410), and 69.3% (199/287) in participants with mild, moderate, moderately severe, and severe to very severe AFL subjects, respectively (Figure 4; *p* = 0.001).

### Factors Related to Non-vaccination in Participants With AFL

As shown in Table 2, in multivariable analyses, younger age [adjusted odds ratio (OR) for being unvaccinated = 0.88, 95% confidence interval (CI) = 0.87–0.90], male (adjusted OR = 1.70; 95% CI = 1.21–2.42), and current smoking (adjusted OR = 1.42, 95% CI = 1.01–2.00) were associated with increased odds for being unvaccinated. Although participants with more severe AFL were more likely to be vaccinated according to the univariable analysis, the severity of AFL was not related to vaccination status in multivariable analyses.

**Table 2:** Factors associated with non-vaccination against influenza in participants with airflow limitation.

**Variables**	**Univariable**	Multivariable^*^
	**OR (95% CI)**	**OR (95% CI)**
Male sex	1.47 (1.29–1.68)	1.70 (1.21–2.42)
Age, year	0.89 (0.88–0.90)	0.88 (0.87–0.90)
Married state	0.43 (0.26–0.73)	2.33 (0.85–6.40)
Medicaid	0.73 (0.56–0.97)	0.85 (0.47–1.52)
Economic activity	2.42 (2.15–2.73)	0.96 (0.75–1.22)
Education beyond high school	2.28 (2.01–2.57)	1.15 (0.91–1.45)
EQ-5D	8.47 (5.21–13.78)	0.57 (0.21–1.50)
Body mass index, kg/m^2^	1.00 (0.98–1.02)	0.97 (0.93–1.01)
Chronic bronchitis	0.81 (0.64–1.02)	0.89 (0.57–1.37)
Current smoker	1.79 (1.55–2.05)	1.42 (1.01–2.00)
**Severity of airflow limitation** ^†^		
Mild (FEV_1_ %pred of >70)	Reference	Reference
Moderate (FEV_1_ %pred of 60–69)	0.92 (0.78–1.08)	1.07 (0.78–1.49)
Moderately severe (FEV_1_ %pred of 50–59)	0.75 (0.60–0.93)	1.14 (0.77–1.69)
Severe to very severe (FEV_1_%pred of <50)	0.66 (0.51–0.85)	1.22 (0.75–1.97)
**Comorbidities**		
Hypertension	0.58 (0.51–0.67)	0.91 (0.72–1.16)
Ischemic heart disease	0.56 (0.41–0.78)	0.80 (0.47–1.34)
Diabetes mellitus	0.82 (0.72–0.93)	0.92 (0.70–1.22)
Asthma	0.51 (0.78–0.68)	0.61 (0.33–1.13)

*Data are presented as ratios (95% confidence intervals)*.

† *Severity of airflow limitation was graded according to ATS/ERS guideline (25), and severe and very severe were combined into one group due to the small number of participants with severe to very severe AFL (n = 64)*.

* *Multivariate analysis was adjusted for age, sex, smoking PY, marriage status (married vs. unmarried or divorced), economic activity (active vs. inactive), education level (college or above vs. high school or less), EQ-5D, severity of airflow limitation, and comorbid conditions (hypertension, ischemic heart disease, diabetes mellitus, and asthma)*.

*OR, odds ratio; EQ-5D, EuroQol-5 dimensions questionnaire; FEV_1_, forced expiratory volume in 1s; % pred; percentage of the predicted value*.

## Discussion

This study evaluated influenza vaccination trends from 2007 and 2018 among participants with AFL in Korea. During the study period, the overall influenza vaccination rate was higher in participants with AFL than in those without AFL. While the influenza vaccination rate in the elderly with AFL steadily increased similar to that of those without AFL, the rate in young participants with AFL decreased, while the rate among young participants without AFL fluctuated. In multivariable analyses, younger age, male, and current smoking were associated with increased odds of unvaccinated status.

Influenza vaccination has been considered as important management in individuals with chronic airway diseases with AFL, such as COPD, asthma, and bronchiectasis. The current guidelines strongly recommend influenza vaccination in COPD. Influenza vaccination is known to reduce not only acute exacerbations but also serious lower respiratory infections in COPD participants. The benefits of influenza vaccination in participants with COPD extend beyond the prevention of respiratory illness. Studies have shown that influenza vaccination can reduce mortality and risk of ischemic heart disease, especially in the elderly (31). Furthermore, chronic comorbidities are highly prevalent in patients with COPD and are associated with frequent exacerbations and increased risk of exacerbation (32). Hence, influenza vaccination should be emphasized in patients with AFL and other comorbidities.

The vaccination rate in patients with COPD varies widely by country. While the rates of influenza vaccination in patients with COPD were relatively high in the US (~64%) (21), Spain (~60%) (17, 18), and Korea (~60% in this study), the vaccination rate was relatively low in Hungary (24%) (19), Taiwan (32%) (33), and Turkey (40%) (20). Unfortunately, few studies have evaluated influenza vaccination coverage within a specific country. In our study, the influenza vaccination rate was relatively stable, suggesting more effort is needed to increase coverage in patients with COPD in Korea. Because the current data suggest that vaccination rates can vary widely, we suggest that studies evaluating vaccination rates over time would be informative to set country-specific vaccination strategies for COPD patients.

Regarding asthma, a previous meta-analysis demonstrated influenza vaccination reduced febrile illness by 72% and prevented 59–78% of cases of acute exacerbation of asthma requiring emergency visits and/or hospitalizations (22), suggesting influenza vaccination could be a cost-effective strategy to reduce acute exacerbation of asthma. Although there is limited evidence directly showing the effectiveness of vaccination in patients with bronchiectasis, influenza virus may play a crucial role in triggering exacerbation of bronchiectasis (34), which supports providing influenza vaccination to this population (23, 24). However, to provide solid evidence for influenza vaccination in patients with bronchiectasis, future studies evaluating the role of influenza vaccination in bronchiectasis are needed.

Importantly, our study results suggested whom clinicians should persuade to receive influenza vaccination, which includes younger patients, males, and current smokers. As shown in previous studies, the vaccination rate was significantly lower in the younger population with AFL than in the older population with AFL (17, 20, 21, 33, 35). Additionally, younger age was independently associated with unvaccinated status in participants with AFL. Beyond this, we further performed a trend analysis for vaccination over 10 years according to age group, which has not been performed in previous studies. While the vaccination rate in elderly subjects with AFL had a steadily increasing trend, the rate showed a decreasing trend. The reasons for this phenomenon are not clear. For a possible reason, young participants with AFL are likely to have unhealthy lifestyles, while their symptoms are mild or absent in the early stage of AFL. Accordingly, the importance of influenza vaccination is likely to be neglected in this population. This information suggests the importance of age in predicting influenza vaccination in those with AFL. We need to pay more attention to increasing the vaccination rate in the young population with AFL.

Consistent with previous studies, our study revealed that males and current smokers are more likely to be unvaccinated among participants with AFL. Generally, males are more likely to be current smokers than females, especially in Asian countries, including Korea (30). Furthermore, being a male and a smoker increases the risk of severe disease presentation, including mortality (36–38). Thus, it is plausible that such male patients who also have AFL have a much higher risk of worse prognosis than those without these risk factors. However, our study results alone cannot explain sex differences in vaccination rates. We believe that an unhealthy lifestyle in men may have influenced this observation. Additionally, there is a possibility that women might be more compliant with clinicians' recommendations for influenza vaccination than men, as shown by the sex difference in medication adherence in Korea (39).

There have been conflicting results on the relationship between AFL severity and influenza vaccination rate. While a lower rate of vaccination was observed in subjects with more severe AFL (17), other studies showed a positive association between AFL severity and influenza vaccination rate (18–20). Similarly, our study showed a positive association between AFL severity and vaccination rate, although it diminished after adjustment of covariables. Given that severe AFL can be associated with severe pneumonia in patients with AFL (40), and the protective effects of influenza vaccination seem to be correlated with AFL severity (13), strategies focusing on improving the vaccination rate in patients with severe AFL might be more cost-effective.

Our findings yield important insights that can be helpful to design targeted strategies to increase influenza vaccination coverage in patients with AFL. The identification of factors (younger age, male, and current smokers) associated with unvaccinated status can help design tailored strategies to increase influenza vaccination in patients with AFL. We also suggest a change in strategy of influenza vaccination policy for adults in Korea. Currently, the Korean government-led free influenza vaccination policy for adults is a one-size-fits-all strategy that is applied to all subjects aged 65 or older, and it does not consider risk factors other than age. Thus, a large number of young patients with AFL who are at a high risk of influenza have not been considered for the benefit of government-led free vaccination programs. However, given the broad benefits of influenza infection on respiratory diseases with AFL, a more advanced and personalized free vaccination strategy that estimates individual risk might be helpful, and it could include young patients with AFL.

There are limitations to our study that should be acknowledged. First, this study was performed in Korea, limiting the generalizability of our findings. Second, because KNHANES does not have data on post-bronchodilator spirometry, we defined AFL using pre-bronchodilator spirometry. However, prebronchodilator spirometry has been widely used to define AFL in many previous studies of COPD epidemiology (30, 41, 42). Third, this study could not evaluate the presence of bronchiectasis, which may be an important comorbidity to consider when interpreting the results of our study. This was because a questionnaire on bronchiectasis was only available in the 2007–2009 NHANES dataset. Fourth, we could not provide a reasonable explanation for our observation of the relationship between socioeconomic and influenza vaccination status in participants with AFL. Poor socioeconomic status, such as low education level, inactive economic status, and reduced QoL, showed increased odds of non-vaccination in the univariable analysis but no significant relationship in multivariable analyses. Although the reasons are not clear, it is possible that the Korean government-led free influenza vaccination program attenuated the influence of these factors on influenza vaccination. The free influenza vaccination program in Korea targets people receiving Medicaid and the elderly population whose socioeconomic status and QoL are poor, which leads to disproportionally higher influenza vaccination rates in these subjects despite their poor socioeconomic status. Thus, the simultaneous consideration of these factors (age, Medicaid, economic activity, educational level, and QoL) might have yielded the non-significance of socioeconomic variables. As socioeconomic status and government health policies might differ between countries, our study results should be interpreted with caution in other countries.

In conclusion, over the past 10 years, the influenza vaccination rate in elderly participants with AFL steadily increased, while the rate in younger participants with AFL decreased. Younger participants, males, and current smokers were most likely to have unvaccinated status among those with AFL. More attention and targeted interventions are required to improve the influenza vaccination rate in individuals with AFL.

## Data Availability Statement

Publicly available datasets were analyzed in this study. This data can be found here: https://knhanes.kdca.go.kr/knhanes/main.do.

## Ethics Statement

This study used data from KNHANES which was approved by the Institutional Review Board of the Korea Centers for Disease Control (IRB No. 1401–047-547). The patients/participants provided their written informed consent to participate in this study.

## Author Contributions

HL and YJ take responsibility for the data, analysis, and wrote the first draft of the manuscript. HL, HC, and YJ designed the study. YJ performed statistical analysis of data. All authors provided critical review and approved the version for publication.

## Funding

This work was supported by the National Research Foundation (NRF) of Korea Grant funded by the Ministry of Science, Information and Communications Technologies (MSIT) (NRF-2020R1F1A1070468), the Bio and Medical Technology Development Program of the National Research Foundation funded by the Korean Government (MSIT) (NRF-2021M3E5D1A01015176), and the Korea Medical Device Development Fund Grant funded by the Korea Government (the Ministry of Science and ICT, the Ministry of Trade, Industry and Energy, the Ministry of Health and Welfare, the Ministry of Food and Drug Safety) (Project Number: 1711138447, KMDF_PR_20200901_0214).

## Conflict of Interest

The authors declare that the research was conducted in the absence of any commercial or financial relationships that could be construed as a potential conflict of interest.

## Publisher's Note

All claims expressed in this article are solely those of the authors and do not necessarily represent those of their affiliated organizations, or those of the publisher, the editors and the reviewers. Any product that may be evaluated in this article, or claim that may be made by its manufacturer, is not guaranteed or endorsed by the publisher.

## References

[1] Mathers CD Loncar D. Projections of global mortality and burden of disease from 2002 to 2030. PLoS Med. (2006) 3:e442. 10.1371/journal.pmed.0030442 17132052 PMC1664601

[2] Mannino DM Braman S. The epidemiology and economics of chronic obstructive pulmonary disease. Proc Am Thorac Soc. (2007) 4:502–6. 10.1513/pats.200701-001FM 17878461

[3] Suissa S. Dell'Aniello S, Ernst P. Long-term natural history of chronic obstructive pulmonary disease: severe exacerbations and mortality. Thorax. (2012) 67:957–63. 10.1136/thoraxjnl-2011-201518 22684094 PMC3505864

[4] McGhan R Radcliff T Fish R Sutherland ER Welsh C Make B. Predictors of rehospitalization and death after a severe exacerbation of COPD. Chest. (2007) 132:1748–55. 10.1378/chest.06-3018 17890477

[5] Dransfield MT Kunisaki KM Strand MJ Anzueto A Bhatt SP Bowler RP. Acute exacerbations and lung function loss in smokers with and without chronic obstructive pulmonary disease. Am J Respir Crit Care Med. (2017) 195:324–30. 10.1164/rccm.201605-1014OC 27556408 PMC5328181

[6] Garcia-Aymerich J Serra Pons I Mannino DM Maas AK Miller DP Davis KJ. Lung function impairment, COPD hospitalisations and subsequent mortality. Thorax. (2011) 66:585–90. 10.1136/thx.2010.152876 21515553

[7] Press VG Konetzka RT White SR. Insights about the economic impact of chronic obstructive pulmonary disease readmissions post implementation of the hospital readmission reduction program. Curr Opin Pulm Med. (2018) 24:138–46. 10.1097/MCP.0000000000000454 29210750 PMC5810972

[8] Wedzicha JA Seemungal TA. COPD exacerbations: defining their cause and prevention. Lancet. (2007) 370:786–96. 10.1016/S0140-6736(07)61382-8 17765528 PMC7134993

[9] Sapey E Stockley RA. COPD exacerbations. 2: aetiology. Thorax. (2006) 61:250–8. 10.1136/thx.2005.041822 16517585 PMC2080749

[10] Papi A Luppi F Franco F Fabbri LM. Pathophysiology of exacerbations of chronic obstructive pulmonary disease. Proc Am Thorac Soc. (2006) 3:245–51. 10.1513/pats.200512-125SF 16636093

[11] Lall D Cason E Pasquel FJ Ali MK Narayan KM. Effectiveness of influenza vaccination for individuals with chronic obstructive pulmonary disease (COPD) in low- and middle-income countries. COPD. (2016) 13:93–9. 10.3109/15412555.2015.1043518 26418892

[12] Ouaalaya EH Falque L Dupis JM Sabatini M Bernady A Nguyen L. Susceptibility to frequent exacerbation in COPD patients: impact of the exacerbations history, vaccinations and comorbidities? Respir Med. (2020) 169:106018. 10.1016/j.rmed.2020.106018 32442114

[13] Chan TC Fan-Ngai Hung I Ka-Hay Luk J Chu LW Hon-Wai Chan F. Effectiveness of influenza vaccination in institutionalized older adults: a systematic review. J Am Med Dir Assoc. (2014) 15:226.e1–e6. 10.1016/j.jamda.2013.10.008 24321878

[14] Kopsaftis Z Wood-Baker R Poole P. Influenza vaccine for chronic obstructive pulmonary disease (COPD). Cochrane Database Syst Rev. (2018) 6:CD002733. 10.1002/14651858.CD002733.pub3 29943802 PMC6513384

[15] Schembri S Morant S Winter JH MacDonald TM. Influenza but not pneumococcal vaccination protects against all-cause mortality in patients with COPD. Thorax. (2009) 64:567–72. 10.1136/thx.2008.106286 19321465

[16] Global Initiative for Chronic Obstructive Lung Disease. Global Strategy for the Diagnosis, Management, Prevention of Chronic Obstructive Pulmonary Disease. Global Initiative for Chronic Obstructive Lung Disease (2021). Available online at: http://www.goldcopd.org/ (accessed December 7, 2021).

[17] Garrastazu R Garcia-Rivero JL Ruiz M Helguera JM Arenal S Bonnardeux C. Prevalence of influenza vaccination in chronic obstructive pulmonary disease patients and impact on the risk of severe exacerbations. Arch Bronconeumol. (2016) 52:88–95. 10.1016/j.arbr.2015.09.022 26526292

[18] Ruiz Azcona L Roman-Rodriguez M Llort Bove M van Boven JF Santibanez Marguello M. Prevalence of seasonal influenza vaccination in chronic obstructive pulmonary disease (COPD) patients in the Balearic Islands (Spain) and its effect on COPD exacerbations: a population-based retrospective cohort study. Int J Environ Res Public Health. (2020) 17:27. 10.3390/ijerph17114027 32517007 PMC7312905

[19] Fekete M Pako J Nemeth AN Tarantini S Varga JT. Prevalence of influenza and pneumococcal vaccination in chronic obstructive pulmonary disease patients in association with the occurrence of acute exacerbations. J Thorac Dis. (2020) 12:4233–42. 10.21037/jtd-20-814 32944335 PMC7475525

[20] Ozlu T Bulbul Y Aydin D Tatar D Kuyucu T Erboy F. Immunization status in chronic obstructive pulmonary disease: a multicenter study from Turkey. Ann Thorac Med. (2019) 14:75–82. 10.4103/atm.ATM_145_18 30745939 PMC6341858

[21] Saeed GJ Valero-Elizondo J Mszar R Grandhi GR Cainzos-Achirica M Omer SB. Prevalence and disparities in influenza vaccination among patients with COPD in the United States. Chest. (2021) 159:1411–4. 10.1016/j.chest.2020.10.058 33129793

[22] Vasileiou E Sheikh A Butler C El Ferkh K von Wissmann B McMenamin J. Effectiveness of influenza vaccines in asthma: a systematic review and meta-analysis. Clin Infect Dis. (2017) 65:1388–95. 10.1093/cid/cix524 28591866 PMC5850022

[23] Hill AT Sullivan AL Chalmers JD De Soyza A Elborn SJ Floto AR. British Thoracic Society Guideline for bronchiectasis in adults. Thorax. (2019) 74:1–69. 10.1136/thoraxjnl-2018-212463 30545985

[24] Polverino E Goeminne PC McDonnell MJ Aliberti S Marshall SE Loebinger MR. European Respiratory Society guidelines for the management of adult bronchiectasis. Eur Respir J. (2017) 50:1700629. 10.1183/13993003.00629-2017 28889110

[25] Pellegrino R Viegi G Brusasco V Crapo RO Burgos F Casaburi R. Interpretative strategies for lung function tests. Eur Respir J. (2005) 26:948–68. 10.1183/09031936.05.00035205 16264058

[26] EuroQol G. EuroQol–a new facility for the measurement of health-related quality of life. Health Policy. (1990) 16:199–208. 10.1016/0168-8510(90)90421-9 10109801

[27] Lee YK Nam HS Chuang LH Kim KY Yang HK Kwon IS. South Korean time trade-off values for EQ-5D health states: modeling with observed values for 101 health states. Value Health. (2009) 12:1187–93. 10.1111/j.1524-4733.2009.00579.x 19659703

[28] National National Cholesterol Education Program (NCEP) Expert Panel on Detection Evaluation and and Treatment of High Blood Cholesterol in Adults (Adult Treatment Panel III). Third report of the national cholesterol education program (NCEP) expert panel on detection, evaluation, and treatment of high blood cholesterol in adults (Adult Treatment Panel III) final report. Circulation. (2002) 106:3143–421. 10.1161/circ.106.25.3143 12485966

[29] American Diabetes Association. Standards of medical care in diabetes−2013. Diabetes Care. (2013) 36(Suppl. 1):S11–66. 10.2337/dc13-S011 23264422 PMC3537269

[30] Lee H Shin SH Gu S Zhao D Kang D Joi YR. Racial differences in comorbidity profile among patients with chronic obstructive pulmonary disease. BMC Med. (2018) 16:178. 10.1186/s12916-018-1159-7 30285854 PMC6171244

[31] Huang CL Nguyen PA Kuo PL Iqbal U Hsu YH Jian WS. Influenza vaccination and reduction in risk of ischemic heart disease among chronic obstructive pulmonary elderly. Comput Methods Programs Biomed. (2013) 111:507–11. 10.1016/j.cmpb.2013.05.006 23769164

[32] Westerik JA Metting EI van Boven JF Tiersma W Kocks JW Schermer TR. Associations between chronic comorbidity and exacerbation risk in primary care patients with COPD. Respir Res. (2017) 18:31. 10.1186/s12931-017-0512-2 28166777 PMC5294875

[33] Huang HH Chen SJ Chao TF Liu CJ Chen TJ Chou P. Influenza vaccination and risk of respiratory failure in patients with chronic obstructive pulmonary disease: a nationwide population-based case-cohort study. J Microbiol Immunol Infect. (2019) 52:22–9. 10.1016/j.jmii.2017.08.014 28927683

[34] Gao YH Guan WJ Xu G Lin ZY Tang Y Lin ZM. The role of viral infection in pulmonary exacerbations of bronchiectasis in adults: a prospective study. Chest. (2015) 147:1635–43. 10.1378/chest.14-1961 25412225 PMC7094490

[35] Hsu DJ North CM Brode SK Celli BR. Identification of barriers to influenza vaccination in patients with chronic obstructive pulmonary disease: analysis of the 2012 behavioral risk factors surveillance system. Chronic Obstr Pulm Dis. (2016) 3:620–7. 10.15326/jcopdf.3.3.2015.0156 27981230 PMC5154688

[36] Martinez A Soldevila N Romero-Tamarit A Torner N Godoy P Rius C. Risk factors associated with severe outcomes in adult hospitalized patients according to influenza type and subtype. PLoS ONE. (2019) 14:e0210353. 10.1371/journal.pone.0210353 30633778 PMC6329503

[37] Wong CM Yang L Chan KP Chan WM Song L Lai HK. Cigarette smoking as a risk factor for influenza-associated mortality: evidence from an elderly cohort. Influenza Other Respir Viruses. (2013) 7:531–9. 10.1111/j.1750-2659.2012.00411.x 22813463 PMC5855151

[38] Godoy P Castilla J Soldevila N Mayoral JM Toledo D Martin V. Smoking may increase the risk of influenza hospitalization and reduce influenza vaccine effectiveness in the elderly. Eur J Public Health. (2018) 28:150–5. 10.1093/eurpub/ckx130 29020390

[39] Jeong H Kim H Lee K Lee JH Ahn HM Shin SA. Medical visits, antihypertensive prescriptions and medication adherence among newly diagnosed hypertensive patients in Korea. Environ Health Prev Med. (2017) 22:10. 10.1186/s12199-017-0619-6 29165108 PMC5664834

[40] Eom JS Song WJ Yoo H Jeong BH Lee HY Koh WJ. Chronic obstructive pulmonary disease severity is associated with severe pneumonia. Ann Thorac Med. (2015) 10:105–11. 10.4103/1817-1737.151441 25829961 PMC4375738

[41] Shin SH Park J Cho J Sin DD Lee H Park HY. Severity of airflow obstruction and work loss in a nationwide population of working age. Sci Rep. (2018) 8:9674. 10.1038/s41598-018-27999-6 29946117 PMC6018711

[42] Kim DS Kim YS Jung KS Chang JH Lim CM Lee JH. Prevalence of chronic obstructive pulmonary disease in Korea: a population-based spirometry survey. Am J Respir Crit Care Med. (2005) 172:842–7. 10.1164/rccm.200502-259OC 15976382

